# A Word with Our Readers

**Published:** 1879-11

**Authors:** 


### A WORD WITH OUR READERS.

We do not like to bring the subject into the columns of the Journal, but having failed to effectively reach many of our subscribers through the ordinary channels of business, we must take this means of saying a few words to them. *We want money*, and from those who owe us. There are many physicians who have been receiving the Journal for three or four years, and have never paid one cent towards its support. It costs money to print the

JOURNAL, and the expense of getting it up must be paid promptly. To pay our obligations promptly we must collect from our subscribers with some degree of promptness. Those who owe for subscriptions are aware of the fact, and we trust that they will make a remittance to the publisher at once. He needs the money, and only asks for what is justly due him.

